# Structural and chemical evolution of Au-silica core–shell nanoparticles during 20 keV helium ion irradiation: a comparison between experiment and simulation

**DOI:** 10.1038/s41598-020-68955-7

**Published:** 2020-07-21

**Authors:** M. Mousley, W. Möller, P. Philipp, G. Hlawacek, T. Wirtz, S. Eswara

**Affiliations:** 1 Advanced Instrumentation for Nano-Analytics (AINA), MRT Department, Luxembourg Institute of Science and Technology, 41, rue du Brill, 4422 Belvaux, Luxembourg; 2 Institute of Ion Beam Physics and Materials Research, Helmholtz-Zentrum Dresden-Rossendorf e.V., Bautzner Landstr. 400, 01328 Dresden, Germany

**Keywords:** Nanoparticles, Scanning probe microscopy, Atom optics

## Abstract

Au-silica core–shell nanoparticles have been irradiated with 20 keV He^+^ ions up to a maximum fluence of 4.7 × 10^17^ ions/cm^2^. The nanoscale structural and crystallographic evolution induced by He^+^ ion irradiation was followed at various stages using Transmission Electron Microscopy (TEM). During irradiation satellite Au clusters are formed around the main Au core, which remained crystalline even after the maximum He^+^ ion fluence. The spherical silica shell deformed into a hemisphere due to He^+^ ion irradiation. Three dimensional Monte-Carlo simulations, based on the binary collision approximation, have been performed on stacked infinite layers and an individual particle. The stacked layers results show that the He^+^ beam interacts with most of the nanoparticle and Au migrates in the direction of beam incidence agreeing with experimental findings. The individual particle results match the experiment in terms of the volume which is sputtered away however additional mechanisms, not included in the simulations, are present in the experiment during the satellite formation and silica shell deformation. These results show the ability for 20 keV He^+^ ions to be used for the modification of nanostructures. Furthermore, these results contribute to a quantitative understanding of the dynamic evolution of materials observed using microscopy techniques based on He^+^ ions.

## Introduction

The helium ion microscope (HIM) uses a scanned He^+^ (or Ne^+^) beam to probe a sample surface, similarly to a scanning electron microscope (SEM). HIM is already a useful technique for analysing surfaces^1–3^. However, the commercially available machines are currently focused on the signals emitted back from the sample, so called secondary signals (secondary electrons^4,5^, secondary ions^6,7^, secondary photons^8,9^). The transmitted signal has received much less attention, however there has been some recent interest^10–13^. Collecting the transmitted signal makes it possible to record information from the entire depth of the sample which is missing in the secondary electron image, due to the small interaction volume from which the secondary electrons can escape. This will help in the investigation of buried structures. A transmission HIM (THIM) also offers the potential for interesting imaging modes and contrast not accessible via a conventional transmission electron microscope (TEM), such as imaging the ions which have been neutralised by the sample. For instance, in a TEM charge exchange between the probe and the sample cannot be directly investigated. In addition, the wavelength of He^+^ is much smaller than an electron with the same energy, thus reducing the diffraction limited distances which can be resolved, offering an improved theoretical resolution limit.

At LIST, a transmission HIM (THIM) prototype instrument has been developed which is capable of recording the transmitted helium ion signal from keV ions in both stationary-beam as well as scanning mode^14^.There is still much work to be done in assessing the capabilities of such THIM systems using sub 50 keV ions, and, in particular, the sample damage by the incident ions. In this paper we investigate the behaviour of Au-silica core–shell nanoparticles under He^+^ irradiation in our THIM prototype.

Au-silica core–shell nanoparticles were investigated due to their possible applications in multiple fields including plasmonics^15^, sensing^16^ and healthcare^17^. It is the optical response which is key to these uses, and this interaction with photons is directly linked to the structure of the particles. This means techniques to investigate the structure of these particles, which THIM can be a part of, are vital for the future progress of this field. Previous studies have investigated the effect of radiation on similar nanoparticle systems. However these have mostly been at MeV energies and with ions heavier than helium^18–21^. The size of the particle has been shown to determine what the beam shaping effect will be, either shaping into nanorods, faceting or no major change^22^.

This study investigates the response of Au-silica core–shell nanoparticles to 20 keV He^+^ ions. The previously reported effects of silica shell deformation (using 4 MeV Xe^+^ ions)^23,24^ and formation of Au satellite clusters (using 4 MeV Au^+^ ions)^22^ are also shown to occur for incident helium at this lower energy. In addition, we show the motion of core–shell particles perpendicular to the ion beam and the formation of a hemispherical structure, both of which were not discussed in the previous studies. Our experimental results are then compared with computational simulations to understand the mechanisms underpinning the nanoscale structural and chemical evolution.

## Methods

Au-silica core–shell nanoparticles were purchased from Sigma Aldrich (product 747,572), each has a Au core of approximately 20 nm diameter surrounded by a 25 nm silica shell. The range of 20 keV helium ions is 62.4 nm for Au and 148 nm for SiO_2_(25 nm)/Au(20 nm)/ SiO_2_(25 nm)/430 nm C. These were calculated by SRIM simulations using monolayer collision steps. This means the helium ions used in this study have enough energy to penetrate through the nanoparticles. The initial solution was diluted with deionised water by a factor of 10 and then shaken by hand. A drop of this solution was placed onto plastic film (Parafilm, Bemic Company inc. ) and, using tweezers, a holey carbon grid (Plano S160-9) was inserted into the bottom of the drop. This was to allow any debris smaller than the nanoparticles to float to the top of the droplet, allowing the nanoparticles to sink onto the grid. This successfully reduced the amount of debris on the grid but it did allow particles to attach to both sides of the grid. After a few minutes the grid was pulled out of the droplet. The side of the grid was touched onto filter paper to wick away most of the water and a filament lamp was used to dry the sample within a few minutes. In this way, it was found that the nanoparticles attached to the carbon with their silica shells intact and without any contamination. Once the sample had dried, the grid was transferred between the TEM and the THIM instrument for TEM imaging and He^+^ exposure respectively.

For the He^+^ ion beam irradiation a THIM instrument was used (details of which have been previously described^14^). Briefly, this THIM comprises of a duoplasmatron ion source operated at 20 keV with helium gas, 2 Einzel lenses, 3 sets of X, Y deflector plates and a microchannel plate coupled to a phosphor screen detector placed behind the sample. A CCD camera is used to record the image on the phosphor screen.

A faraday cup is mounted below the sample and, as such, the beam current could not be measured during the irradiation. The faraday cup current will not include any neutrals hitting the cup, however prior measurements have shown that the neutrals from the source are 1% of the intensity and so we can use the measured ion currents as reliable estimates of the incident fluence.

To account for fluctuations in the beam current a video was recorded during the exposure. The beam current was recorded before and after the irradiation. These start and end current values were equated to the intensity at the initial and final frames of the CCD video. The intensity of the video image, within the area of interest was then averaged over the exposure time and this average intensity was converted to an estimated average beam current using a linear interpolation between the beginning and end faraday cup current values. To calculate the area, the freehand selection tool in ImageJ^25^ was used to measure the exposed area from the CCD video (see “Supplementary information”). The area and beam current estimates then gave the estimated fluence measurements in Table 1. The final fluence was reached by increasing the beam current density by focusing the beam onto the sample. This meant that only an extra 2 h 35 min were required to reach five times the fluence which previously took over 5 h.

**Table 1:** The details of the ion irradiation fluences for the Au core-silica shell particles.

Exposure	Fluence (ions/cm^2^) [1 × 10^14^ /cm^2^ = 1/nm^2^]	Estimated ion total hitting particle	Estimated ion total hitting core
i	0	0	0
ii	6.6 × 10^14^	2.7 × 10^4^	1.4 × 10^3^
iii	9.4 × 10^15^	3.8 × 10^5^	1.9 × 10^4^
iv	1.1 × 10^17^	4.3 × 10^6^	2.2 × 10^5^
v	4.7 × 10^17^	1.9 × 10^7^	9.6 × 10^5^

During the irradiation the sample chamber pressure was around 1 × 10^–7^ mbar. The TEM images indicate that there is no noticeable hydrocarbon deposition on the nanoparticles even after extended ion irradiation at this chamber pressure.

For the TEM imaging a modified FEI Tecnai F20 (as described in^26^) was used. To limit any damage induced by the electron beam it was operated at 120 keV and the magnification was kept to a maximum of 80,000 times. For these imaging conditions no electron beam induced degradation was observed. A collection of particles were imaged by TEM before ion irradiation and then after each subsequent fluence. Four different exposures were performed, details of which are shown in Table 1.

These fluence values were chosen because previous experiments have shown that values between 1 × 10^14^ ions/cm^2^ and 1 × 10^17^ ions/cm^2^ cover different degrees of defect formation in a substrate under irradiation of He^+^ at energies of 7 keV to 35 keV^27^.

**Figure 1:**
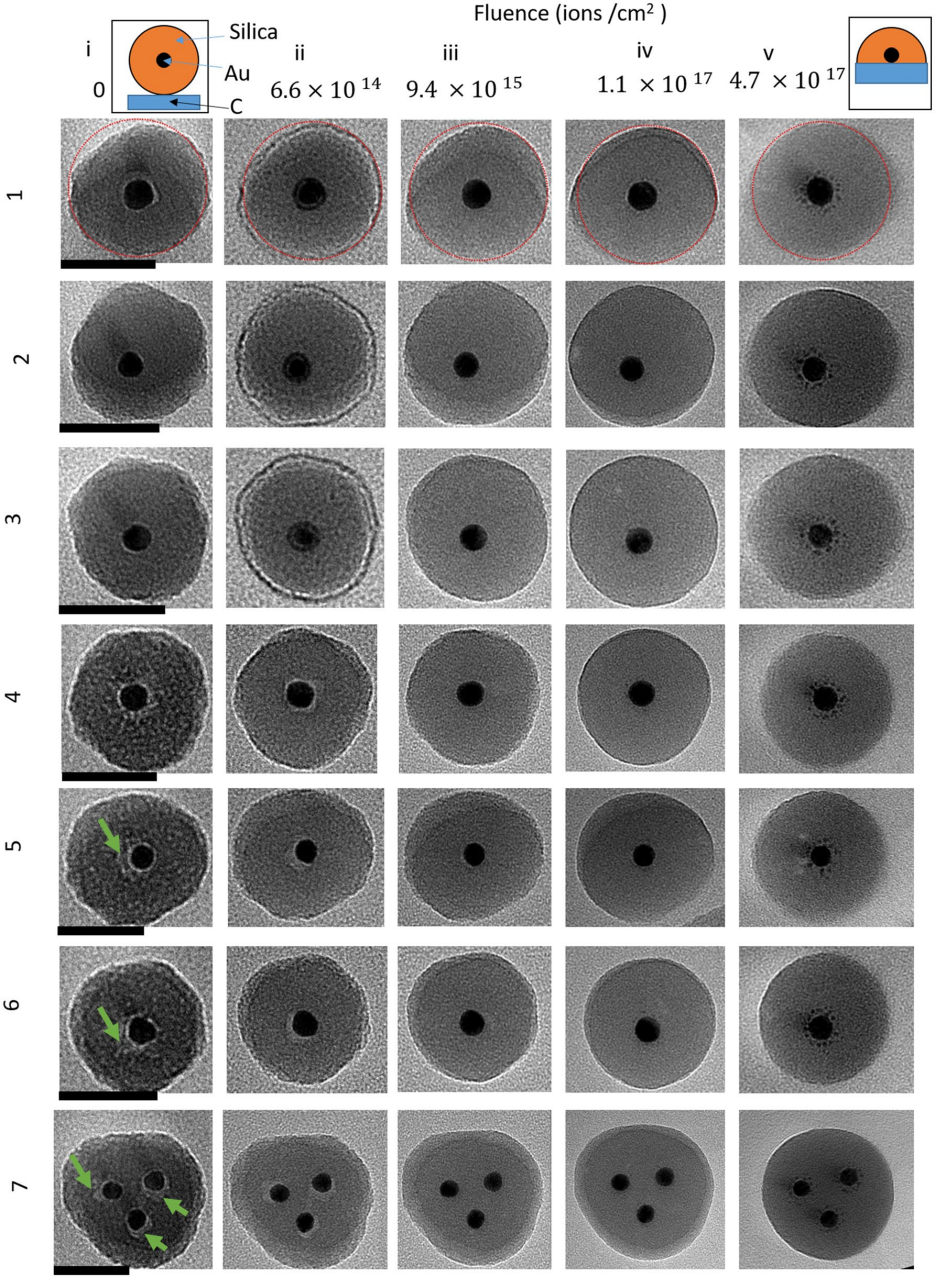
TEM images of nanoparticles (1–7) exposed to increasing fluences (i–v). Scale bars in column i are 50 nm and apply to each row respectively. Particles 1–5 were below the substrate and particles 6 and 7 were above the substrate. ‘Above’ and ‘below’ are relative to the incident ion (and electron) beam travelling from the top (above) to the bottom (below) of the substrate. Schematics at the top of columns I and v show the deformation of the particle as viewed from the side. Green arrows in column 1 show some artefacts ascribed to cavities.

For the computer simulation of the ion irradiation effects, two types of dynamic Monte Carlo codes have been employed, which both make use of the binary collision approximation (BCA). All simulations do not include temperature effects and so are effectively at 0 K. SDTrimSP was used for a 3D simulations of flat surfaces, where only infinite layers of material can be created meaning it is only possible to define a 1D distribution of elements^28^. This software is based on the simulation codes TRIM^29,30^ and TRIDYN^31,32^ but presents the advantage of including an option to take into account the outgassing or diffusion of atoms in the sample^33^. In the present work, this option was applied to helium. The parameter for diffusion is kept identical to previous work^34–36^. Although the diffusion coefficient in the core–shell particles is certainly lower than in polymer samples, TEM imaging gives no evidence for helium accumulation in the nanoparticles. Whilst cavities can be associated with trapped helium, the cavities that were observed before irradiation (see Fig. 1) disappeared during the experiment and thus these are not due to trapped helium. Hence, overestimating the helium diffusion somewhat will have no impact on the conclusions drawn from the simulations. 20 keV helium irradiation was modelled using SiO_2_/Au/SiO_2_ layers on a carbon substrate. The layer thicknesses of 25 nm for SiO_2_ and 20 nm for Au corresponds to the dimensions through the particle centre. The carbon substrate has a thickness of 430 nm, leading to an overall thickness of 500 nm subdivided into 1,000 layers of 5 Å. A fluence of $5\times {10}^{17}$ ions/cm^2^ was simulated in 100 steps using $5\times {10}^{5}$ pseudoparticles per fluence step. Compared to TRI3DYN, 3D information is missing but the relatively high number of pseudoparticles allowed for a good precision of the Au concentration in the silica shell and for some good statistics on collision cascade properties and sputtering. Incidence angles of 0°, 45°, 75° and 85° have been chosen to model the irradiation at the centre of the particle, and at different locations towards the outer edge. The Kr–C potential^37^has been used for interatomic interactions, the Oen–Robinson^38^ model for electronic stopping and the Gauss–Mehler method with 16 pivots for integration. The surface binding energy is calculated using $sb{e}_{i}=0.5\times \left(E{s}_{i}+E{s}_{j}\right)$, where $sbe$ is the surface binding energy for the combination of atoms i and j, and $E{s}_{i}$ and $E{s}_{j}$ are the atomic surface binding energies. The subscripts i and j can take the values for He, Au, Si and O^28^. For SDTrimSP the displacement energies are the default values taken from the internal table (Au: 36 eV; Si: 13 eV; O: 0.5 eV; C: 25 eV).

For fully 3D simulations with the present core–shell nanospheres, TRI3DYN^39^ has been applied. The collisional algorithms are similar as in SDTrimSP, here with electronic energy loss being modelled as an equipartition of local^38^ and nonlocal^40^ interaction. For the surface sputtering of the SiO_x_ compound, composition-dependent surface binding energies have been chosen with parameters which reproduce the sublimation enthalpy of Si at x = 0 and which result in a best fit of high-fluence TRIDYN^31,41^ results to experimental data for sputtering of SiO_2_ by Ar ions in the keV range^42,43^.The amount of atomic displacements and thereby the ion mixing results depend on the choice of the displacement threshold energy. Tabulated data are available for crystalline materials at low damage levels^44–46^ . However, most materials are highly damaged under ion irradiation at high fluences and sufficiently low temperatures. Then, displacements may occur at lower energies due to trapping at pre-existing defects. In their pioneering theoretical paper on ion mixing, Sigmund and Gras-Marti^47^ propose a threshold energy of 7.8 eV for cascade mixing in Si. Consistent with this choice, a default setting of ${U}_{d}=$ 8 eV has been successful in numerous ion mixing, preferential sputtering and thin film deposition studies using TRIDYN^41^, and is also applied here.

TRI3DYN works with a fixed 3D grid of cuboidal voxels (with a volume of 1 × 1 × 1 nm^3^ in the present simulation) which span a cuboidal computational volume. On the voxel grid, arbitrary initial structures can be defined, and their modification of the local bulk composition and surface contour during irradiation can be traced. In order to avoid artificial trapping of ions by the voxel structure at glancing incidence (in particular at the sides of the spheres), a special algorithm replaces the voxelated surface by a locally planar one.

The ballistic transport of incident projectiles or recoil atoms generated in the collision cascades may remove atoms from specific voxels due to bulk displacements or surface sputtering, or add displaced atoms. The incorporation of He projectiles is neglected as its contribution to the local atomic densities is negligible in the present range of ion fluences. For the dynamic relaxation of the structure during irradiation, each moving atom (“pseudoatom”) in the simulation represents a certain number of real atoms, which may be fractional (5.1 in the present), and which is automatically chosen to minimise the computation time whilst retaining sufficient statistical quality of the dynamic development of the system. After a certain number of incident pseudoprojectiles , the dynamic relaxation of the system is activated. In each modified bulk voxel, the total atomic density is re-established by material exchange between neighbouring voxels and transport from/to surface voxels (for details, see ref^48^). Surface voxels which are depleted due to sputtering are combined and partially turned into vacuum voxels. Each relaxation procedure is terminated by an algorithm of surface smoothing.

In the simulations, random broad beam illumination was applied to Au-silica core shell nanoparticles on a carbon membrane with dimensions similar to those used in the experiment. The final fluence was 5 × 10^17^ ions/cm^2^ for which the number of impacts is expected to be similar to the estimates in Table 1.

## Results

As is shown in Fig. 1 there are clear structural changes visible during the irradiation, however these are most noticeable for the fluences iv and v (above 9.4 × 10^15^ ions/cm^2^). For some particles, around the Au core there is initially an area of lighter contrast. The relative positions of these areas were not affected by the lens focus and so are not due to scattering from the Au core, instead these are attributed to cavities inside the silica shell. These cavities are no longer visible in the TEM images after a fluence of 9.4 × 10^15^ ions/cm^2^ (column iii).This shows the silica shell can rearrange to fill the cavity. For some particles there were bright spots inside the silica away from the core (see Fig. 1, particle 5 column v), these changed position with objective lens focus and were indicative of diffraction and scattering from a crystalline Au core.

The silica shells in Fig. 1 also show brighter areas, in a crescent or ring shape, with boundaries that visibly move towards the outer edge of the silica shell during the He^+^ exposure.

**Figure 2:**
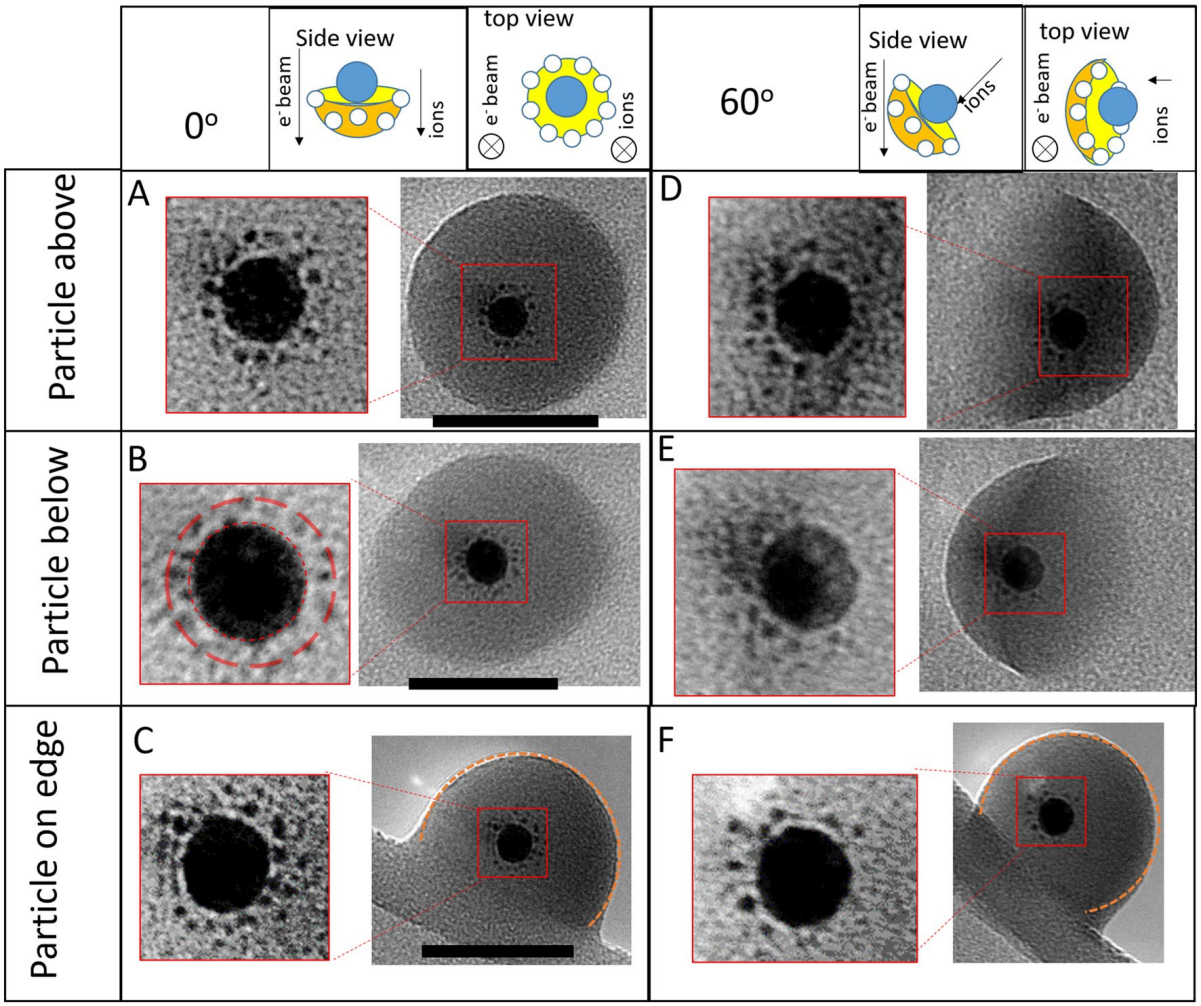
A comparison between 0° tilt (**A**–**C**) and 60° tilt (**D**–**F**) showing the hemispherical shape of particles after 4.7 × 10^17^ ions/cm^2^. The upper row shows schematics of the relative orientation of the sample, the electron beam and the ion beam. The images in the red boxes are magnifications of the regions marked by the red squares, with altered contrast to highlight the satellite positions. The dashed red circles in **B**, show the estimated core surface and average satellite distance. Black scale bars for 0° are 50 nm.

This brighter area is where the particle curves up away from the substrate. A near identical contrast can be seen when tilting the particle, see Fig. 2, and shows the reverse process as the angle is increased, i.e. the crescent boundary moves inwards. The outward motion of the boundary represents a change in the structure to more of a hemispherical dome shape, which can be viewed in images with a 60° tilt after the final irradiation (examples are shown in Fig. 2). This is in contrast to the previously reported deformed silica particles on a silicon substrate (^23,49^) which did not spread over the substrate and maintained their curved surface at the interface with the substrate after irradiation.

For the experimental conditions used and assuming uniform energy distribution, our calculations suggest that the energy deposited by electronic stopping per incident helium ion would result in a temperature rise of 40 K and 2 K for Au (20 nm sphere) and silica shell (25 nm layer between 10 and 35 nm radius). As the heat dissipation rate is unknown, determination of the steady-state temperature is not feasible. To explain the shape change from sphere-like to dome-like two mechanisms could be used. The first would be localised heating of a volume, or surface layer, of the silica shell and subsequent flow of this viscous layer, minimizing surface tension. This is similar to the melting discussed previously for Au-silica core–shell nanoparticles^50^. The large mismatch in the coefficients of thermal expansion between Au and silica would result in tensile stress of silica shell, further enhancing mass transport. Silica shell have previously been shown to break up due to thermal stress whilst heating silica encapsulated bismuth nanoparticles^51^. Besides coalescence, viscous flow can also explain the disappearance of internal cavities seen in pristine particles (cf. Fig. 1). The second mechanism would be excitation of individual atoms into excited surface states , which can then diffuse across the ‘cold’ surface, coming to rest at the connection between the particle and substrate. Both of these mechanisms have been considered before in ion sculpting experiments^52,53^ .Our data are insufficient to choose one or the other, so we present these possible mechanisms and leave the question of which is occurring for future projects.

**Figure 3:**
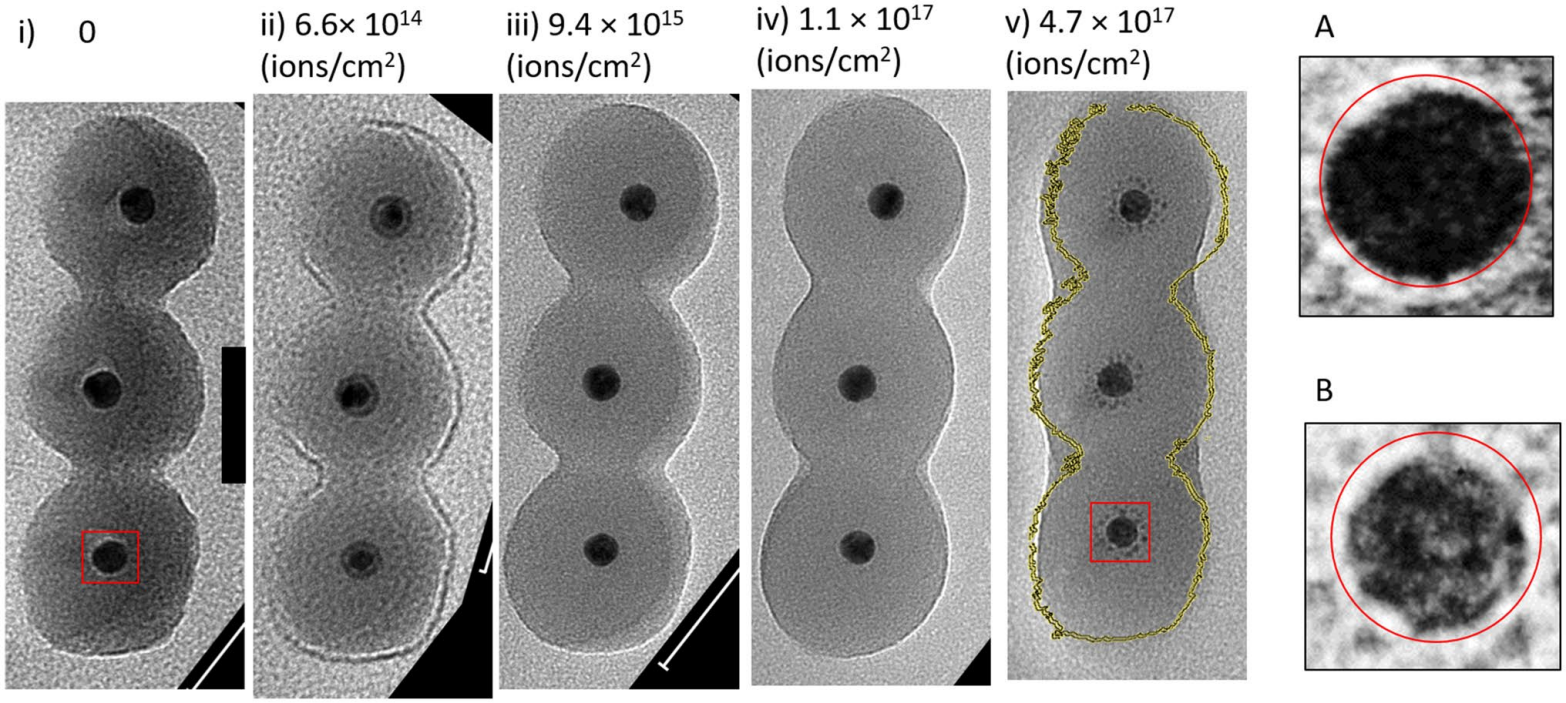
(i–v) TEM images of neighbouring particles after the different fluences in Table 1. A and B show enlarged images of the lower core in the red boxes of i and v respectively. The red rings are identically sized and are guides for the eye. The scale bar in i is 50 nm and applies to i–v . The yellow lines in v are the outlines of the initial particle sizes from figure i.

Regardless of the mechanism causing the deformation, it is clear a hemisphere is formed. The hemispherical shape could be more stable due to the minimisation of the surface area of the silica shell. This argument also offers an explanation for the visible mixing of the row of three particles in Fig. 3.

The silica shells appear to have coalesced together after the final fluence of 4.7 × 10^17^ ions/cm^2^, also reducing the area of the silica-vacuum interface. Coalescence of nanoscale silica has been previously reported in in-situ TEM heating experiments^51,54,55^ Our results show coalescence of silica can be induced by ion-irradiation.

To fill in the grooves between neighbouring particles, the material must come from the surrounding silica shell. For both the individual particle and the chain of three, the radius remains approximately constant during the irradiation (see red circles in Fig. 1 and yellow outline in Fig. 3, as guides for the eye) which can be explained by a balance between sputtering and rearrangement to a more dome like structure. It can also be seen that the relative position of the Au cores has moved within the silica shells, to end up closer to the substrate. This means that during irradiation either the silica shell can move around the Au core, or the Au core can move within the silica shell. From the image, neither the shell shapes nor the relative positions of the cores are stationary and so a combination of both mechanisms is likely.

It can be seen that the silica shell boundary becomes more rounded which can also be explained by rearrangement to a more energetically favourable circular structure. The rounding is particularly clear when comparing the images from the start (Fig. 1 column i) and 1.1 × 10^17^ ions/cm^2^ (Fig. 1 column iv). Previous experiments with MeV ions have seen a deformation of silica particles from spherical to ellipsoidal shapes^56,57^. This was due to an effect called ion hammering which has been explained by a fast thermal spike causing expansion perpendicular to the molten cylindrical ion track in the particle which is then frozen in during rapid cooling^19^. Comparing the 0° and 60° tilt images of the particle on an edge in Fig. 2 , it can be seen that there is limited deformation parallel to the beam. When compared to circular curvature (the dashed arcs) the particle has not really been flattened. This shows the ion hammering effect is not large for these experimental conditions, otherwise a more elliptic profile would be expected when viewing the particle at a 60° tilt.

Another process which could be occurring is some form of intermixing of the carbon substrate with the silica shell , similar to what has previously been simulated^58^. This is supported by the visible reduction in contrast between the edges of the particles and the substrate. Intermixing can also explain some of the merging behaviour visible when a particle is on the edge of the substrate, as shown in Fig. 2 . Here too there is a low contrast between the particle and substrate.

It is worth noting that an additional possible mechanism which could be happening is a burrowing effect, similar to what has been seen for ion irradiation of Pt particles on SiO_2_^59^ and Au particles on SiO_2_^21^. In addition, the alternative process of SiO_2_ particles burrowing onto a composite metal surface has been shown to occur after laser irradiation^60^. The burrowing is due to the minimisation of surface energy and, for complete burrowing, this requires a bulk substrate which is thick enough to cover all of the particle surface. However, here the carbon film is around 10 nm and is covered on the bottom side with a formvar layer of around 10 nm giving a total substrate depth of around 20 nm, which is less than the 30 nm radius of the particle Fig. 1. This means the membrane is not thick enough for the particle to burrow completely into the layer. If the particles have burrowed, then, when the sample is tilted, one would expect to see some contrast within the membrane, coming from the buried part of the sphere. The particles investigated did not show this contrast in the membrane and so we can infer that the presence of an intact buried hemisphere is rare for the fluences applied. It will be shown later on that burrowing is also inconsistent with the sputtering expected from simulations and the constant radius observed experimentally. We conclude that burrowing is not the dominant deformation mechanism which is occurring.

Particles close to the edge of the carbon membrane, also deformed slightly towards a hemispherical structure, see Fig. 2 and “Supplementary information”. In summary, the best explanation for the observed deformations is a combination of rearrangement of the silica shell (induced by heating or excited surface atom diffusion) and intermixing with the carbon substrate.

During the irradiation, it was seen that some of the nanoparticles had moved across the substrate surface. This could be seen either by the position close to an edge or the relative position of neighbouring particles. This motion could be due to a slight angle between the incident ion and the nanoparticle, caused by a carbon substrate which is not perfectly flat. This could allow some momentum transfer, in the substrate plane. Alternatively, there could be some property of the surface which provides a potential energy where energy minimisation drives the motion. This motion was not visible for all the particles and so the exact cause is unclear.

In addition to the deformation of the silica shell and motion of the particle, there is clear removal of material from the Au core to form satellite clusters of Au around the core. A and B in Fig. 3 show enlarged images of the core before and after irradiation, with a circle overlapped as a guide for the eye, there is a clear reduction in size. The satellite clusters are most visible in Fig. 1, column v, however some faint satellites are also already visible in Fig. 1, column iv too. Similar satellites are known to form after MeV heavy ion irradiation of Au particles^21,61^, and satellites have also been seen during the 10 keV He irradiation of Pt particles on silica^59^. This effect has previously been explained by “inverse” Ostwald ripening which occurs under ion bombardment^18,62^. This is a non-equilibrium scenario in which a negative capillary length means that the concentration around a smaller cluster is lower than larger clusters. Diffusion effects mean that under ion bombardment, the formation and growth of smaller clusters occurs at the expense of the main core. Figures 1,2 and 3 clearly show this is occurring for 20 keV He^+^ irradiation of the nanoparticles in this paper. By a comparison of the tilted and non-tilted views of the satellite structures (Fig. 2) it can be seen that majority of the satellites structures are produced in the direction of beam irradiation, agreeing with previous findings^16,48^. This makes sense when considering that the relocation of recoil atoms preferentially involves the transfer of momentum in the direction of ion incidence. This is not trivial but the displacement of Au recoil atoms is dominated by head on collisions and our simulations (shown below) show that lateral scattering of the ions is only a minor effect. This means the satellites are grouped in a shell underneath the particle, as shown in magnifications of Fig. 3. In addition, there is a visible gap between the shell of satellite clusters and the core, similar to the self-organised layers discussed by Heinig et al.^62^. The gap arises because of competition between migration towards the satellite and migration back towards the larger core. Any atoms ejected from the core to a position between the core and satellite will go to one or the other.

The tilted views in Fig. 2 also demonstrate that particles have attached to both sides of the membrane. The assignment of above or below the sample was done using knowledge of the tilt direction in the microscope and the incident beam direction relative to the sample. This does not appear to affect the irradiation effects seen, all the particles are deformed in the same way and satellites appear in all of the particles. However, the direction of the satellite production is along the direction of ion incidence, for particles above the sample the satellites are pushed towards the flat surface of the dome (i.e. the substrate) for those below the membrane the satellites are formed towards the curved edge, away from the membrane.

Whilst the ion irradiation is capable of deforming the silica shell and Au core, the Au retains crystallinity, as shown by nanoscale TEM Selected Area Electron Diffraction (SAED) patterns (see “Supplementary information”). In addition, looking at column v for particle 5 in Fig. 1, there are clear bright spots near the Au core which move position when the objective lens focus is changed, indicative of diffractive scattering. This has also been reported for Au in silica when irradiated with MeV Au ions^18^.

**Figure 4:**
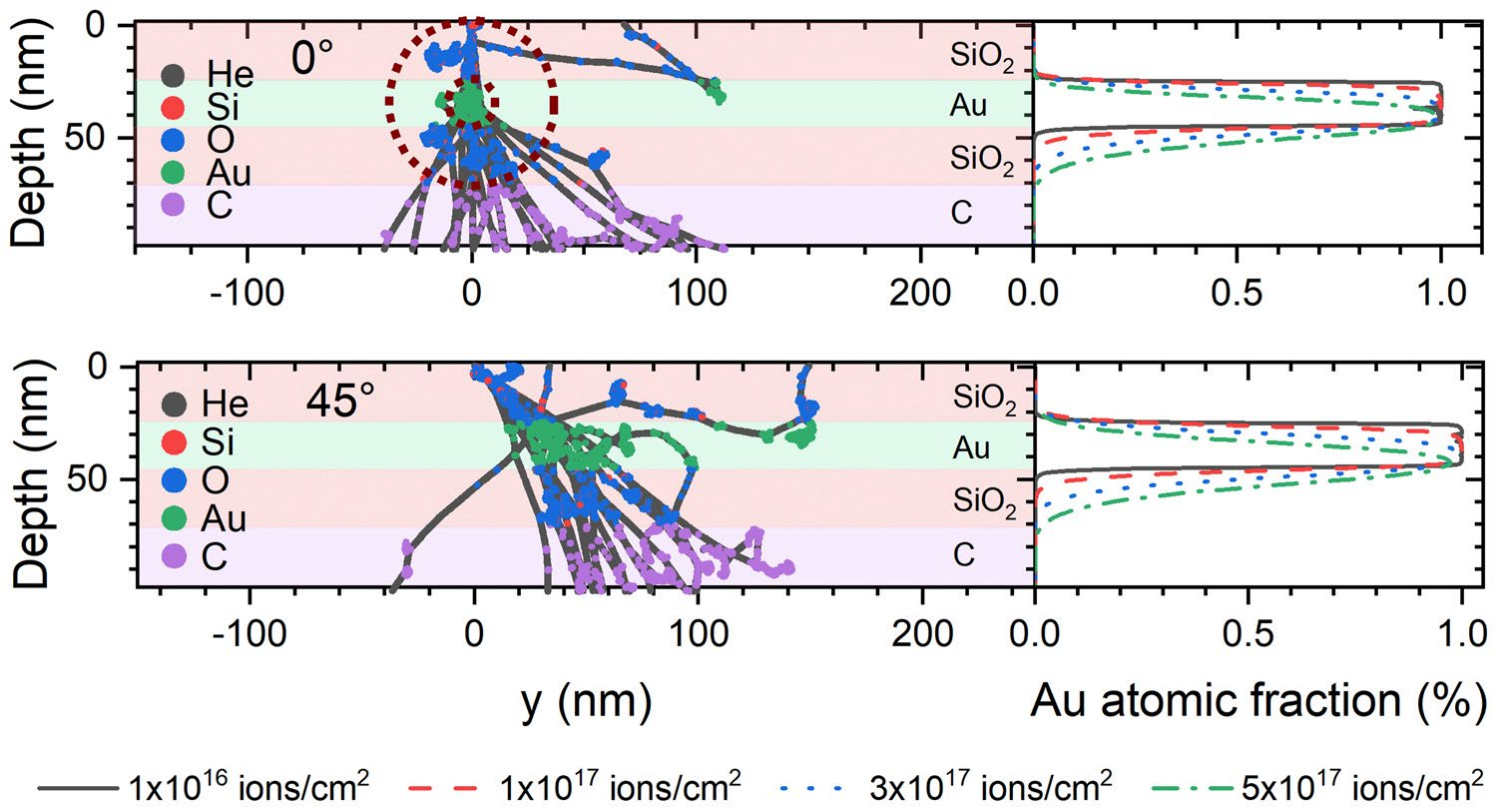
The results of SDTrimSP simulations for two different impact angles (0° and 45°) showing the collision cascades formed by 20 impacts and the Au atomic fraction as a function of depth for a fluences up to 5 × 10^17^ ions/cm^2^. The dashed red circles is a visual aid to compare the size of collision cascade to the size of a particle with a 20 nm core surrounded by a 25 nm thick shell.

To gain a deeper understanding of the processes involved in this ion-particle interaction we have performed simulations of helium ions incident on a layered structure as described in the methods section. The Au atomic fraction profiles in Fig. 4 show intermixing of the Au into the silica layer for the experimental conditions, agreeing with our initial interpretation of the experimental image. The intermixing is into the lower silica layer, in the direction of incident ions, showing Au has been ‘pushed’ out of the central layer, agreeing with the experimental finding of satellite precipitation in the same direction as the He^+^ were travelling. Collision cascades are shown in Fig. 4, these are traces of individual trajectories of atoms each colour showing a different element. Thus an incident helium ion forms a long grey line with multiple different coloured lines coming off it representing those atoms it has displaced. These cascades show that for the elements and dimensions used in this study 20 keV He^+^ spreads out significantly, compared to the outer diameter of the core/shell particle. This means beam broadening is expected and the incident ions can interact with a large proportion of the core/shell particle, as shown by the dashed red outlines in Fig. 4.

Rizza et al. have estimated the threshold atomic density for satellite formation of Au in silica as 2.8 × 10^21^ atoms/cm^3^^18^ , corresponding to an atomic fraction of 11%. The distribution of experimental distances from core surface to satellite centre, from TEM images, was found to peak between 2.5 nm and 3.5 nm (see “Supplementary information”).

The SDTrimSP simulations also showed intermixing of the carbon substrate into the lower silica layer (see “Supplementary information”), meaning this is likely to be occurring during ion irradiation and, as mentioned previously, can partially explain the structural changes in the surface of the particles touching the carbon layer (i.e. the dome shape in Fig. 2).

**Figure 5:**
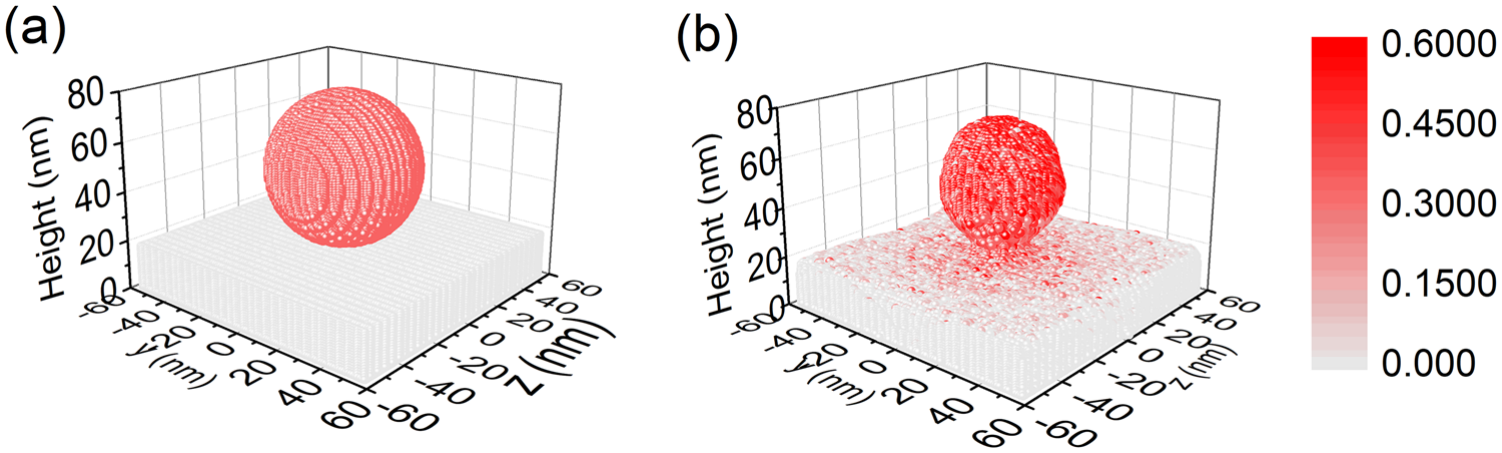
TRI3DYN simulation of composition and shape evolution during irradiation of a 60 nm diameter Au-SiO_2_ core–shell nanosphere on a carbon substrate. (**a**) initial model system; (**b**) system after irradiation with 20 keV He ions from top at a fluence of 5 × 10^17^ cm^-2^. Only surface voxels are shown. The colour represents the Si atomic fraction.

To further understand the processes involved in the fluence-dependent core–shell particle deformations, 3D dynamic Monte Carlo simulations were done using TRI3DYN. Simulations were done for both an individual particle and an infinite chain of particles (by the application of periodic boundaries). To recreate the experimental conditions the energy of the incident ions was 20 keV, and the dimensions were taken from the recorded TEM images which were slightly smaller than the dimensions stated by the manufacturer. As such the simulations used an Au core of 15 nm in diameter and a silica shell outer diameter of 60 nm, the nanosphere being placed on a carbon substrate (see Fig. 5a).

**Figure 6:**
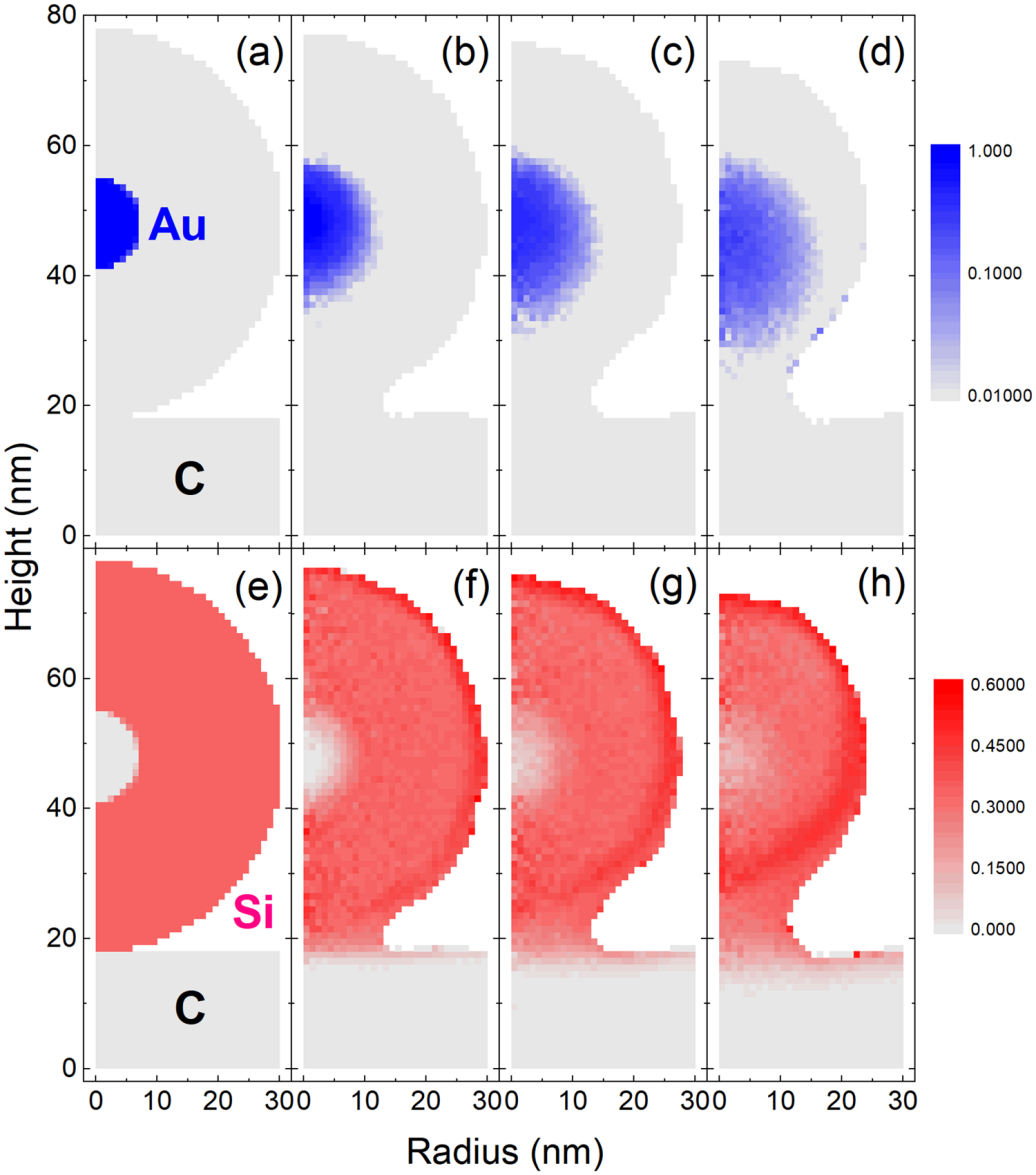
Axial-radial plots of the evolution of shape and atomic fraction distributions of Au (**a**–**d**) and Si (**e**–**h**) initially (**a**,**e**) and after irradiation at fluences of 1 × 10^17^ ions/cm^2^ (**b**,**f**), 2.5 × 10^17^ ions/cm^2^ (**c**,**g**) and 5 × 10^17^ ions/cm^2^ (**d**,**h**), as simulated by TRI3DYN. Note the logarithmic colour scale for the Au distribution.

Irradiation to a fluence of 5 × 10^17^ ions/cm^2^ reduces the size of the nanoparticle significantly (see Figures Fig. 5b and Fig. 6h), which is attributed to surface sputtering. Compared to the top of the particle, more material is removed at the lateral circumference of the body, as the oblique ion incidence there increases the sputtering yield, which is confirmed by our SDTrimSP simulations for different incidence angles. Thus, the original sphere is shaped towards a prolate ellipsoid. The horizontal radius reduces to around 0.8 of its original size, meaning the volume is reduced to around half of the original volume. In contrast, a change in structure to a hemisphere is observed experimentally without a significant alteration of the radius. The latter increases slightly during irradiation as can be seen in Fig. 1 where the yellow outline in the top row is the initial particle outline. This roughly constant radius seems to be due to a coincidental match between the rate at which the silica flows and the sputtering rate at which silica leaves the particle. Nevertheless, the sputter-induced volume loss predicted by TRI3DYN agrees with experimental results.

Figures 5 and 6 demonstrate the formation of a neck between the particle and the substrate by ion-induced material transport from the nanoparticle body towards the substrate. For a simulated chain of particles (see “Supplementary information”) only very narrow transient bridge connections are predicted in contrast to the almost rod-like structures found in the experiments (see Fig. 1). These discrepancies can be explained by the purely collisional nature of the present simulations, which neglect any-potentially ion-induced—effects of surface diffusion and plastic flow, by which the system would tend to minimise the free surface area.

**Figure 7:**
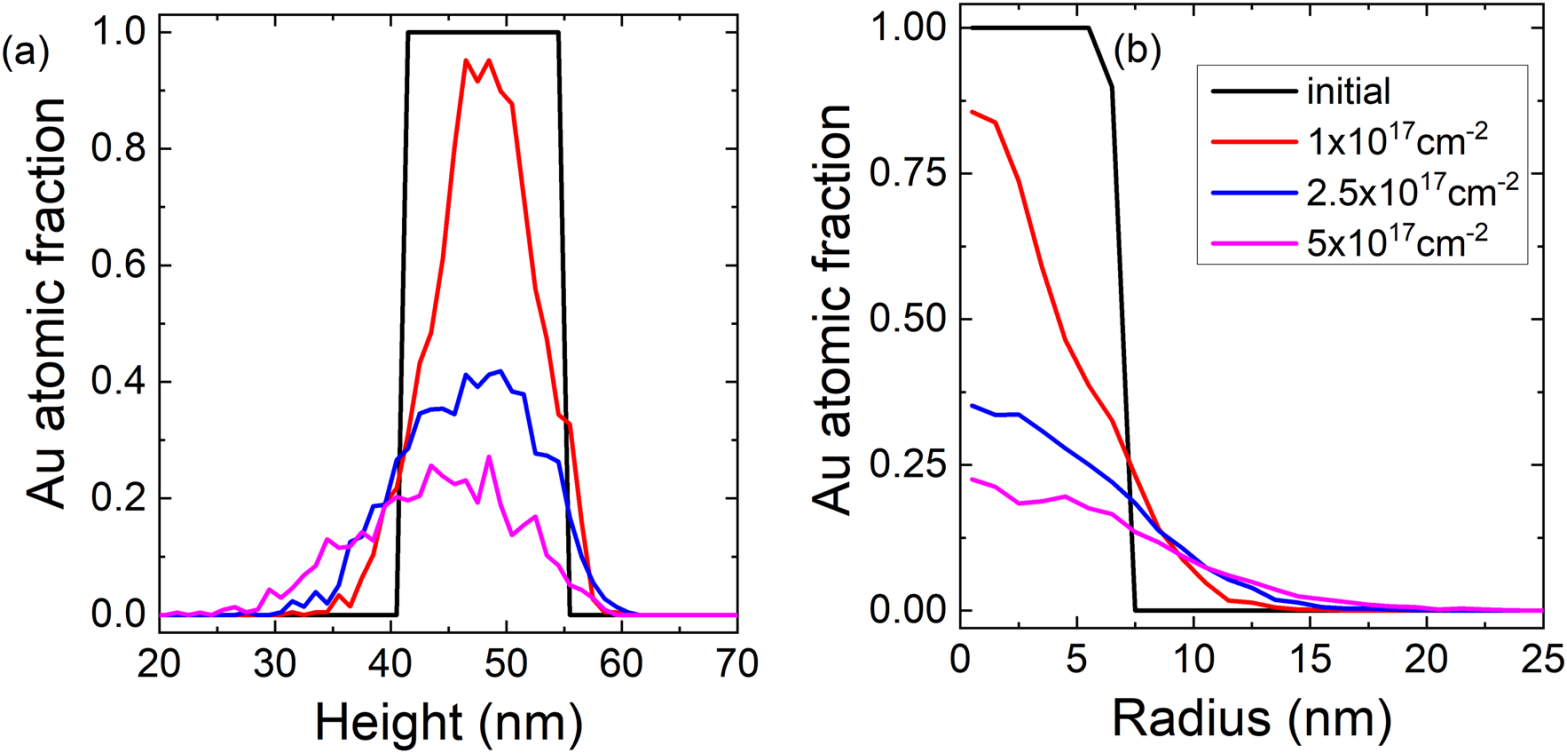
Axial (**a**) and radial (**b**) line plots of the Au atomic fraction, at the fluences indicated by the legend in (**b**), as obtained from TRI3DYN simulation. (**a**) data averaged over a vertical cylinder of 5 nm diameter; (**b**) data averaged over a height range of 4 nm centred at heights of 48 nm (initial), 46 nm (10^17^/cm^2^), 44 nm (2.5 × 10^17^/cm^2^) and 42 nm (5 × 10^17^/cm^2^), thus accounting for the downward shift of the distribution as seen in Fig. 6a–d.

As shown in Fig. 6 there is clear intermixing of the Au core and Silica shell which increases with increasing fluence. Apparently, this increased concentration of Au around the original core leads to the formation of satellite clusters. The simulation does not include any mechanisms for precipitation or thermal diffusion which means that the satellite clusters are not formed in the simulation and the Au simply remains as a low concentration. Consequently, the Au core is also no longer intact after 5 × 10^17^ ions/cm^2^ in contrast to the experiment, showing that similar mechanisms of precipitation act to maintain the core. However, the simulations clearly indicate a downward shift of the Au distribution, which confirms that the Au recoil atoms are preferentially transported by near head-on collisions with the incident He ions. This explains the experimentally observed preferential formation of the satellite nanoclusters around the lower half of the original core with respect to the incident beam (see Fig. 2). For better quantification, Fig. 7 presents axial and radial line plots of the distributions obtained from the simulation. As stated above, the critical Au atomic ratio for satellite precipitation is close to 10%. At a distance of 2–3 nm from the original Au core where the satellites form according to the experiments, this critical concentration is reached above a fluence of around 2 × 10^17^ ions/cm^2^ in both axially forward and radial directions, which is in good agreement with the experimental observations.

Figure 6f–h demonstrates an enrichment of Si both at the shell surface, which is attributed to preferential sputtering of oxygen (see “Supplementary information”), and in a bulk zone close to the bottom of the nanoparticle. The latter is ascribed to preferential forward relocation of oxygen in a depth regime where the damage induced by the incident beam is maximum. Variation in surface composition, caused by preferential sputtering, can also explain variations in sputtering yield with fluence (see “Supplementary information”). From the static TRI3DYN simulation, the mean damage levels at the maximum fluence of 5·10^17^ He/cm^2^ are about 30 dpa and 10 dpa in the Au core and the SiO_2_ shell, respectively. It should be noted that the damage in the shell varies both in lateral direction due to reduced ion penetration at the circumference and in normal direction due to scattering.

Finally, the simulation results show that there is some intermixing of substrate carbon atoms and the silica shell, meaning this also contributes to the experimental deformations. In summary, the simulations do not fully reproduce the experimental details as they solely cover effects which are due to collisional transport. The differences between simulations and experiment prove that additional mechanisms of diffusion, precipitation and plastic flow are active. However, their detailed discussion would exceed the scope of the present paper.

During this experiment, satellite formation was visible for estimated total incident ions of 4.3 × 10^6^ ions on the whole particle. Considering that for standard HIM imaging operating with a beam current of 1 pA and a spot diameter of 1 nm this corresponds to a dwell time as small as 2.4 × 10^–14^ s. This shows that for future STIM experiments, imaging these type of core–shell particles with low energy He^+^ will be challenging. The annular dark-field STIM experiments by Woehl et al.^13^ used 25–30 kV He^+^ on similar Au-silica particles and deformations of the silica shell are visible in their figures. Low beam current densities will be required, in order to avoid the structural changes described here.

## Conclusions

Irradiation of Au-silica core–shell nanoparticles on a carbon support membrane has been reported. 20 keV He^+^ causes structural and chemical modification to both the core and shell. A fluence of 6.6 × 10^14^ ions/cm^2^ caused only slight changes, however for 9.4 × 10^15^ ions/cm^2^ and above there is a prominent deformation of the silica shell into a hemispherical shape along with sputtering of the Au core, forming satellite clusters. The deformation of silica can be explained by either heating and viscous flow of the silica or diffusion of excited surface atoms. Motion of the entire core–shell structure across the surface was also visible after irradiation, showing possible transfer of momentum from the incident ions. One dimensional Monte Carlo simulations reproduced the intermixing expected from experiment as well as the movement of Au out of the core, along the beam direction. 3D Monte Carlo, binary collision approximation simulations using TRI3DYN, showed intermixing of the Au core and the silica shell, deformation of the silica shell, connection with neighbouring particles and intermixing of the silica shell and carbon membrane. The sputter-induced volume loss predicted by TRI3DYN agrees with experimental results. However, to describe other observations such as the satellite formation and silica shell deformation, processes like surface tension, viscous flow and diffusion need to be considered. Any future investigation of similar particles in a THIM will need to consider these effects when deciding on the exposure time. Depending on the application, the deformations could be either undesirable (hindering imaging) or desirable (nanoscale patterning). Future STIM experiments analysing similar particles will require very low beam current densities or cryogenic temperatures if the effects presented in this paper are to be limited. However, we have shown that, whilst previous experiments relied on much higher energies, 20 keV He^+^ can be employed successfully to induce structural changes and create nanoscale satellite clusters.

## Supplementary information

Supplementary information
