# Supplementary material for: Structural and chemical evolution of Au-silica core–shell nanoparticles during 20 keV helium ion irradiation: a comparison between experiment and simulation
Source: Sci Rep. 2020 Jul 21;10:12058. doi: 10.1038/s41598-020-68955-7 (PMC7374165; doi:10.1038/s41598-020-68955-7)
Supplement: Supplementary file 1 — Supplementary information [file 41598_2020_68955_MOESM1_ESM.docx]

**Supplementary information for ‘ Structural and chemical evolution of Au-silica core-shell nanoparticles during 20 keV helium ion irradiation: a comparison between experiment and simulation ’**

M. Mousley^1^, W. Möller^2^, P. Philipp^1^, G. Hlawacek^2^, T.Wirtz^1^, S. Eswara^1^

^1^ Advanced Instrumentation for Nano-Analytics (AINA), MRT Department,

Luxembourg Institute of Science and Technology, 41, rue du Brill, L-4422 Belvaux, Luxembourg

^2^ Institute of Ion Beam Physics and Materials Research, Helmholtz-Zentrum Dresden-Rossendorf e.V., Bautzner Landstr. 400, Dresden, 01328, Germany

Satellite analysis in Imagej

Using Image j the threshold was adjusted such that the inner Au core was black and the surrounding satellites were also included. If necessary sometimes additional cuts were made to separate the inner core from a satellite when the edges were too close (see satellites 10,13 and 17 below). Otherwise the software would assume they are to be treated as one entity when the goal is to measure the central points of the two separately. After the threshold has been applied the ‘Analyze particles’ function was used to get the pixel positions of each satellite and the main core. The distances from the central core surface were then calculated. The core is approximated as a circle of equivalent area, to give the estimated core radius. This radius is then subtracted from the center to center separation to give the estimated distances from the core surface to the satellite centre.


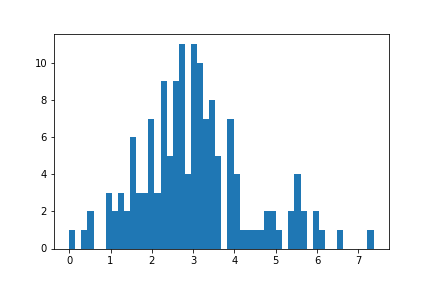


A

B


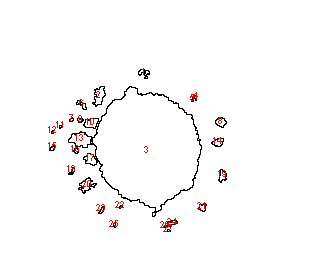


Frequency

Core surface-satellite separation (nm)

Figure 1 An analysis of the satellite distributions using a particle analysis macro (‘analyze’ > ‘analyze particles…’) in Imagej A) the outlined particles from one core, B) the distribution of distances from the core surface (approximated as a circle of equivalent area) to the satellite centres, collected from 5 different particles. The distribution peaks at a surface-satellite separation of around 3 nm.


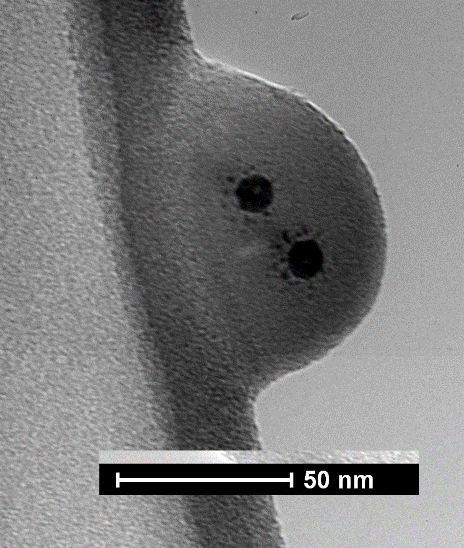


B


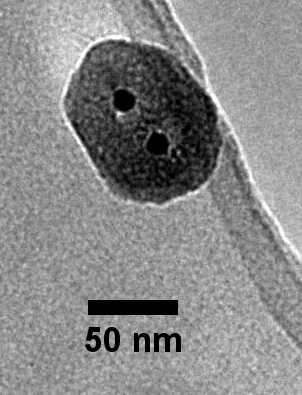


A

60 ^o^ tilt 4.7×10^17^ ions/cm^2^

0 ^o^ tilt 0 ions/cm^2^

Figure 2 - An example of a dual core particle before (a) and after (b) irradiation. Motion of the particle towards the edge and the formation of a hemispherical structure are both clearly visible. Scale bars are 50 nm.


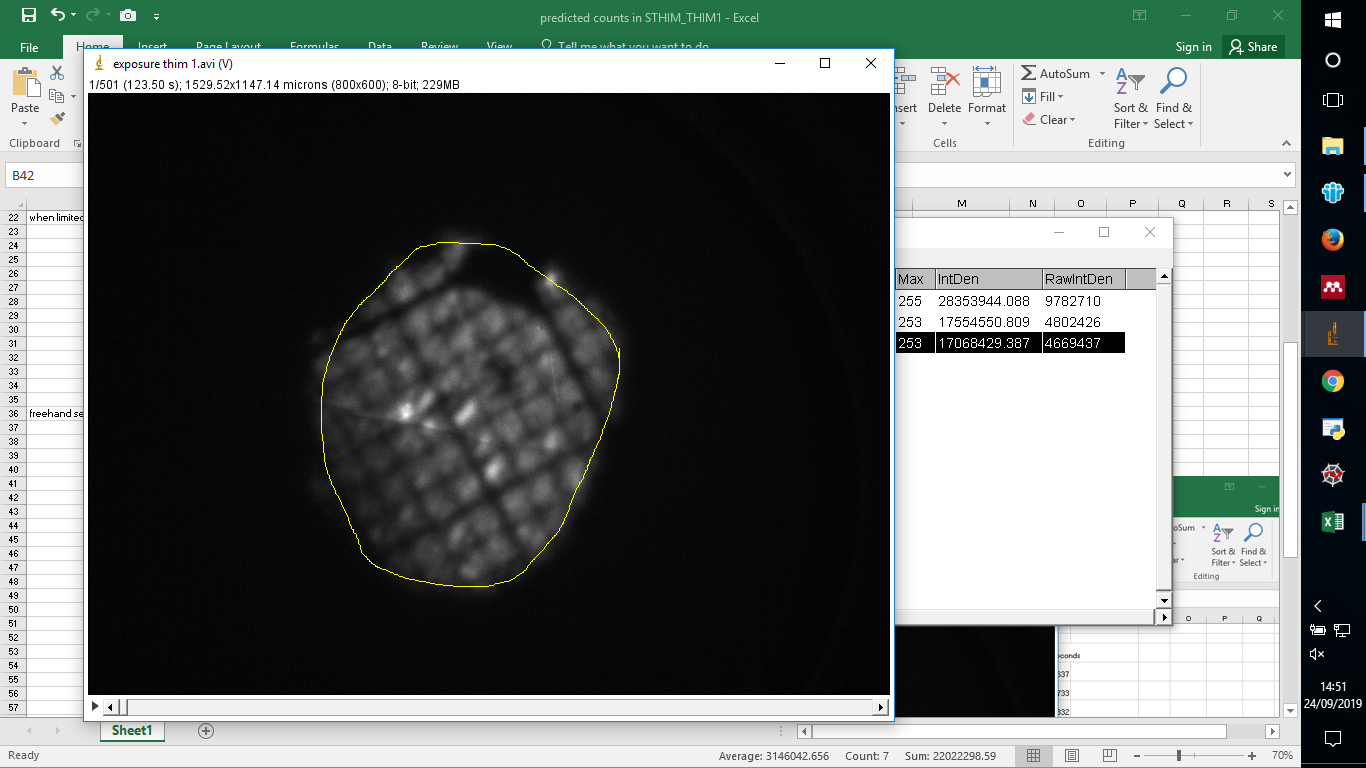


Figure 3 An example of the freehand selection tool being applied to a CCD image of the illuminated area.


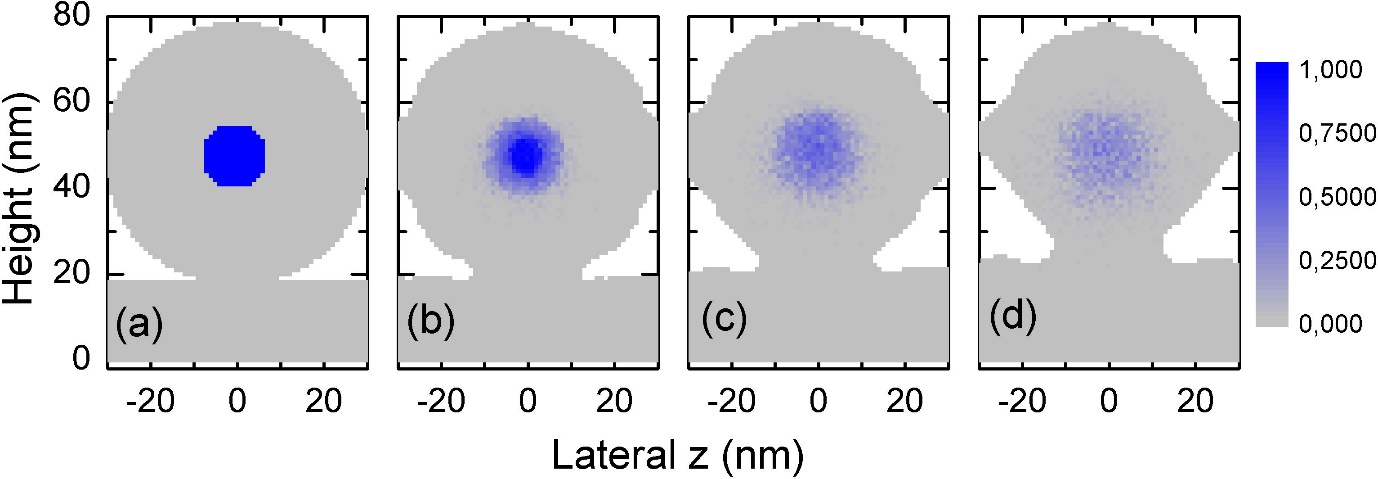


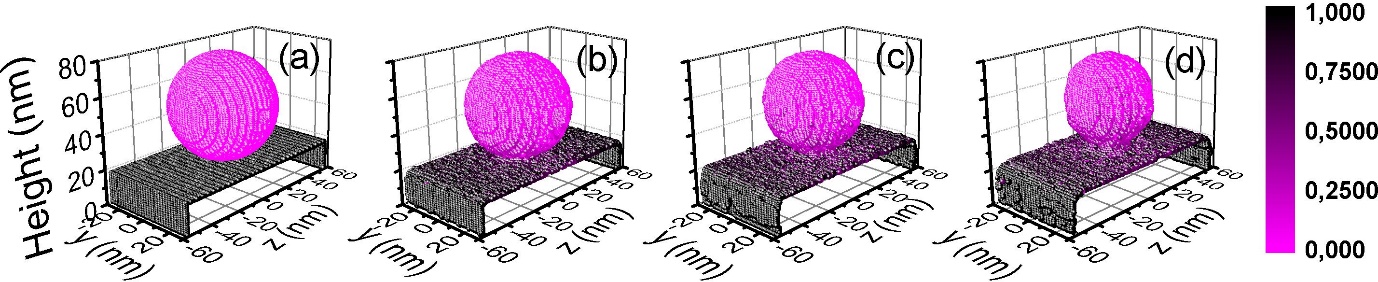


Figure 4 Results from TRI3DYN simulating an infinite chain of nanoparticles on a C substrate initially (a) and after irradiation with 20 keV He^+^ at fluences of 1×10^17^ ions/cm^2^ (b), 2.5×10^17^ ions/cm^2^ (c) and 5×10^17^ ions/cm^2^. Periodic boundary conditions have been applied in the y direction. Upper row: Au atomic fraction averaged over a central slice of 2 nm thickness; lower row: surface voxels with colours according to the carbon atomic fraction. Both the formation of a connection between neighbouring particles and intermixing of the carbon and silica are visible.


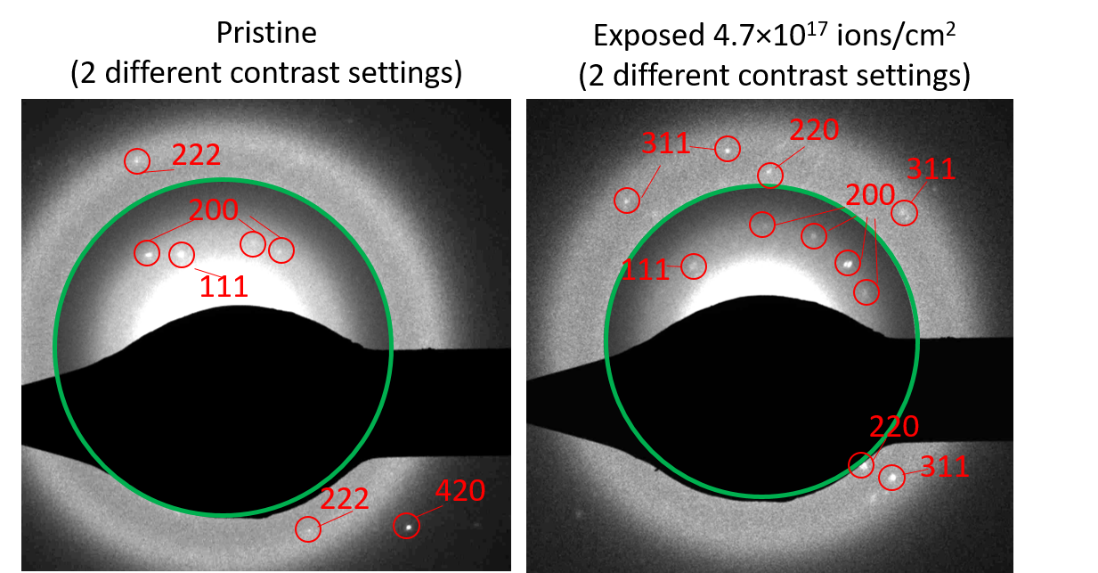


Figure 5 TEM diffraction patterns (log intensity) of the Au-silica core-shell nanoparticles before and after irradiation. The green circle marks the boundary between two different contrast levels used to visualise the diffraction spots. The exposed particle still shows clear diffraction spots.


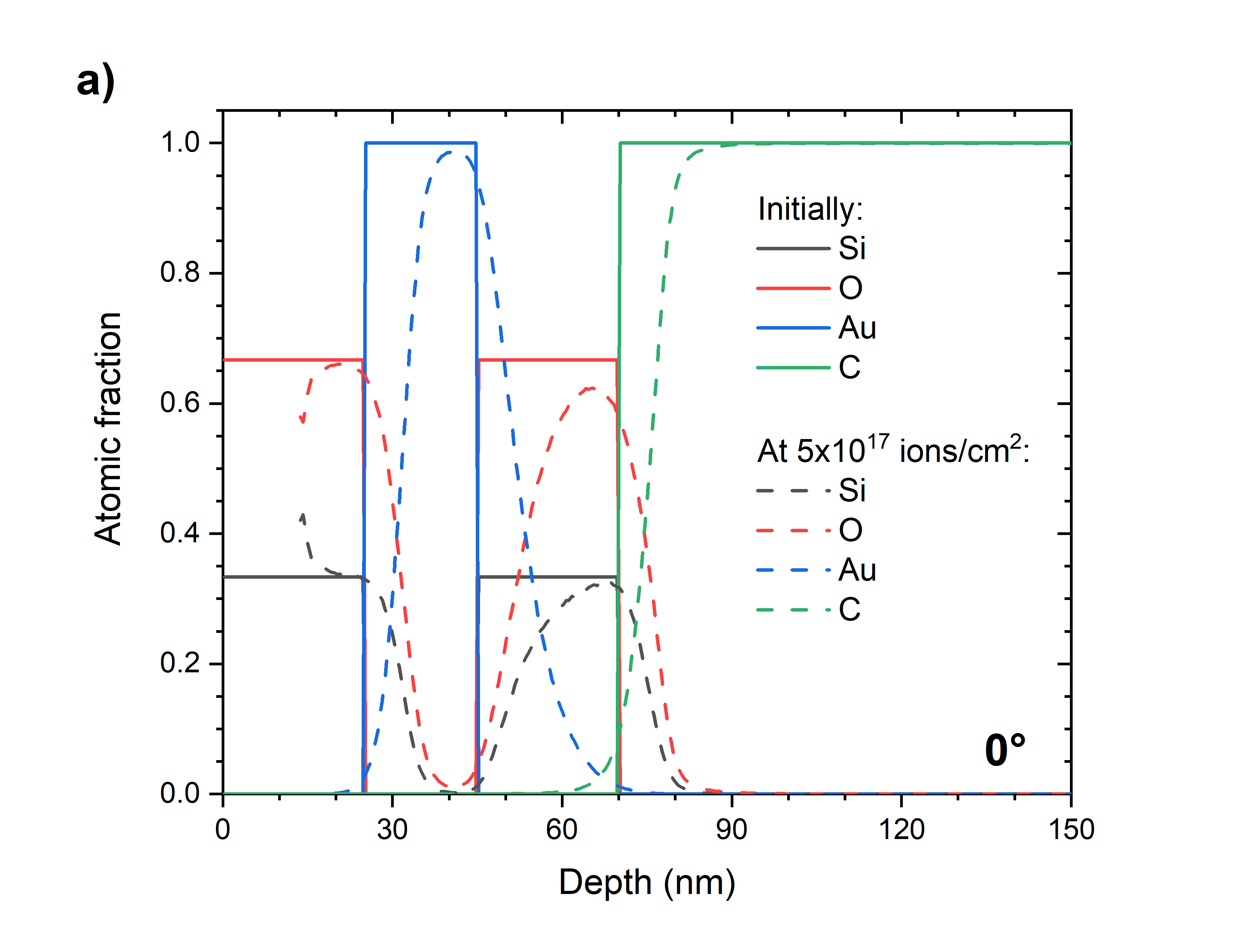

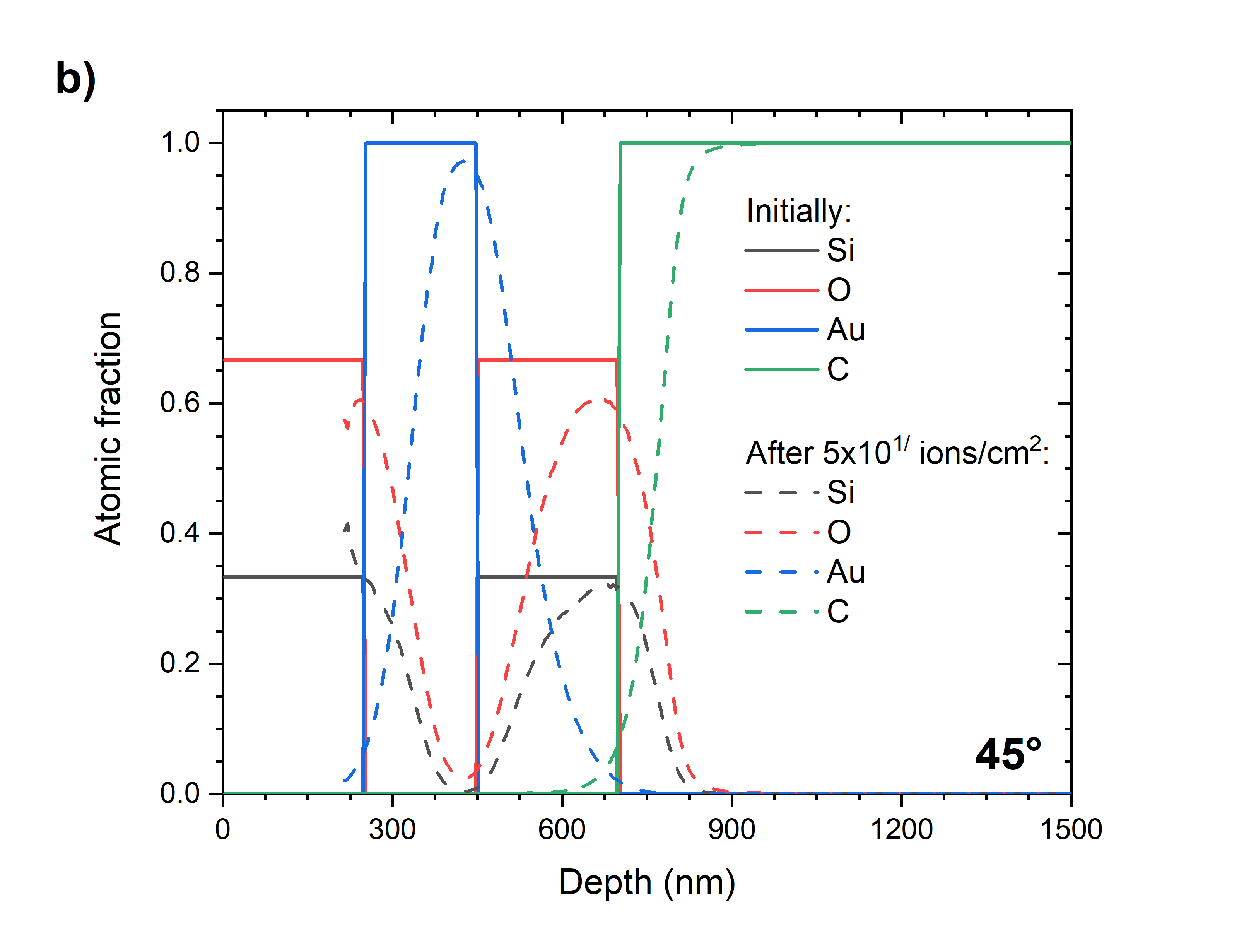


Figure 6 Results from SDTRIMSP simulation, atomic fraction profiles after irradiation with 5×10^17^ ions/cm^2^. For both incidence angles, there is clear intermixing of the carbon into the silica layer.


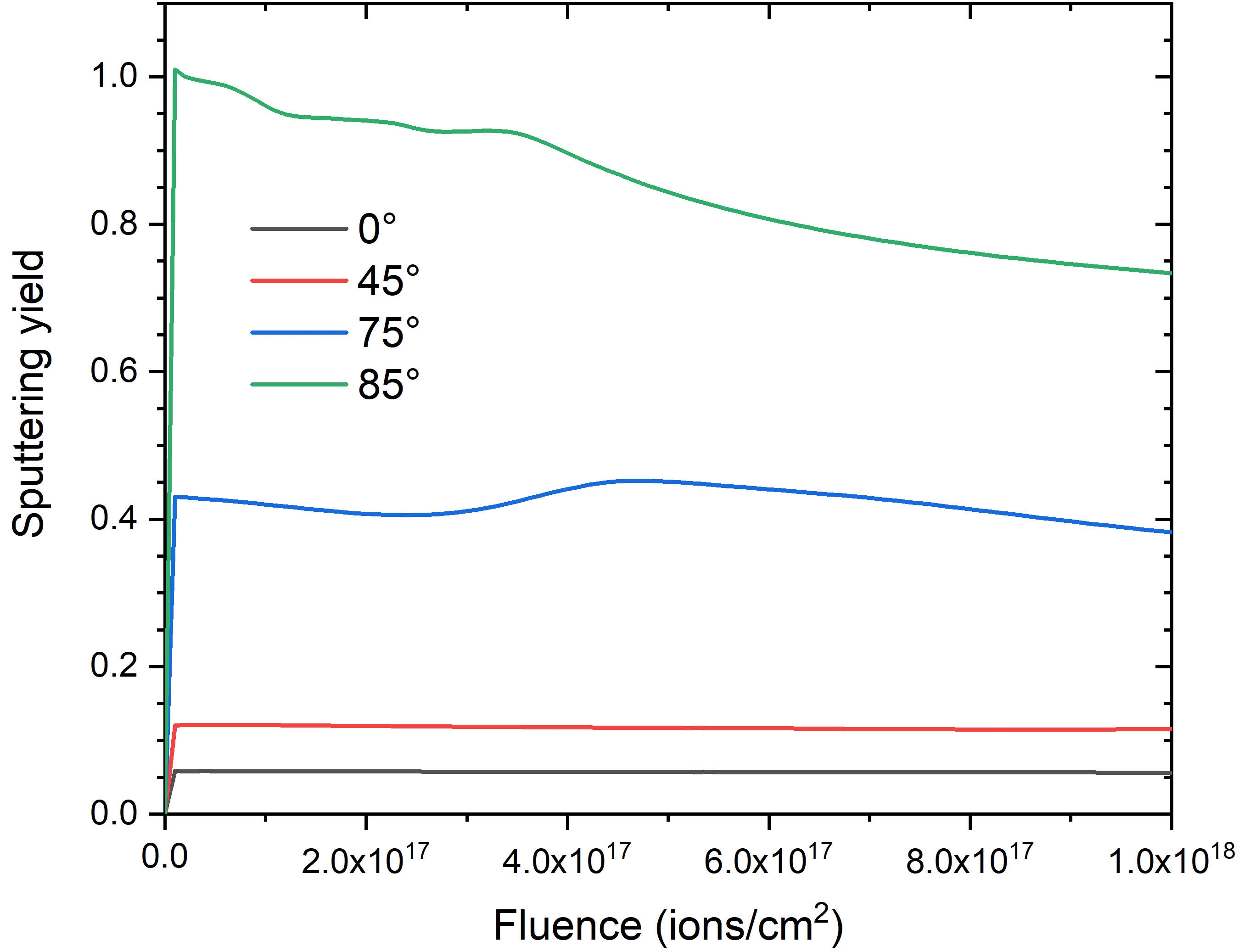


Figure 7 Sputtering yields from the SDTRIMSP simulations, the sputter yield increases for the higher incidence angles.


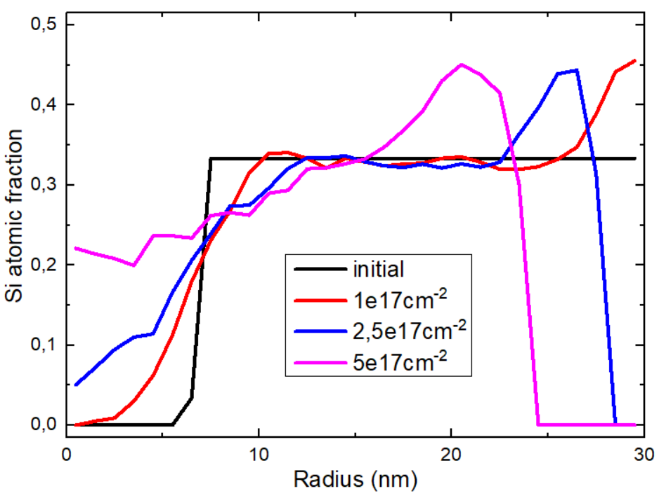

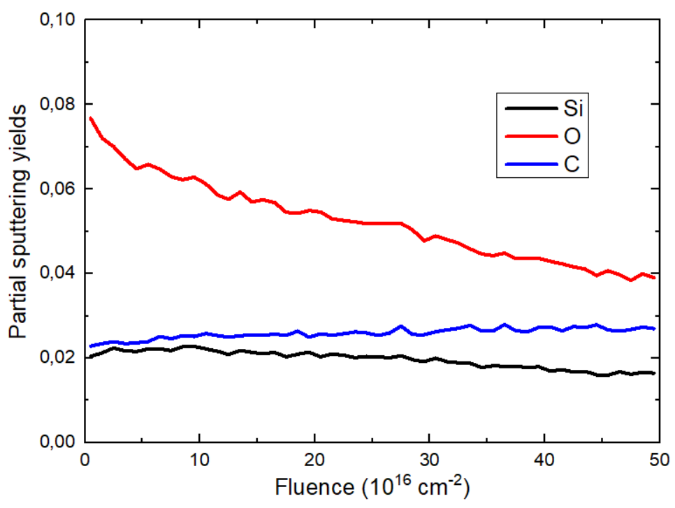


A

B

Figure 8 A) Sputtering yield vs fluence B) Radial profiles of Si at increasing fluence, averaged over a height range of 47 nm to 50 nm
